# CLEC3B as a potential diagnostic and prognostic biomarker in lung cancer and association with the immune microenvironment

**DOI:** 10.1186/s12935-020-01183-1

**Published:** 2020-04-01

**Authors:** Jiaxing Sun, Tian Xie, Muhammad Jamal, Zhenbo Tu, Xinran Li, Yingjie Wu, Jingyuan Li, Qiuping Zhang, Xiaoxing Huang

**Affiliations:** 1 Department of Blood Transfusion, Zhongnan Hospital of Wuhan University, Wuhan, China; 2 Department of Immunology, School of Basic Medical Science, Wuhan University, Wuhan, China; 3 School of Biomedical Sciences, Li Ka Shing Faculty of Medicine, The University of Hong Kong, Hong Kong, China; 4 Department of Pathology, Zhongnan Hospital of Wuhan University, Wuhan, China

**Keywords:** CLEC3B, Lung cancer, Prognosis, Diagnosis, Immune infiltration

## Abstract

**Background:**

Lung cancer is the leading cause of cancer-related mortality globally. Discovering effective biomarkers for early diagnosis and prognosis is important to reduce the mortality rate and ensure efficient therapy for lung cancer patients. C-type lectin domain family 3 member B (CLEC3B) has been reported in various cancers, but its correlation with lung cancer remains elusive.

**Methods:**

The GEO, TCGA and Oncomine databases were analyzed to examine the expression of CLEC3B in lung cancer. The CLEC3B mRNA levels in 15 patient tissue samples were detected by real-time PCR and the CLEC3B protein levels in 34 patient tissue samples were detected by immunohistochemistry. A Chi-square test was performed to analyze the correlation of CLEC3B expression and clinicopathological factors. The diagnostic value of CLEC3B was revealed by receiver operating characteristic (ROC) curves. Univariate and multivariate Cox proportional hazards regression models and Kaplan–Meier plots were used to evaluate the prognostic value of CLEC3B in lung cancer. The TIMER database was used to evaluate the correlation of CLEC3B and immune infiltration. Gene set enrichment analysis revealed tumor‐associated biological processes related to CLEC3B.

**Results:**

CLEC3B is significantly downregulated in lung cancer patients compared with nontumor controls according to database analysis and patient tissue sample detection (p < 0.001). Specifically, CLEC3B is significantly downregulated in stage IA lung cancer patients (p < 0.001) and has a high diagnostic accuracy (area under the receiver operating characteristic curve > 0.9). Moreover, low expression of CLEC3B is related to poor progression-free survival (HR = 0.60, 95% CI 0.49–0.74, p = 8.3e−07) and overall survival (HR = 0.66, 95% CI 0.58–0.75, p = 2.1e−10), indicating it as a risk factor for lung cancer. Multivariate analysis value showed that low expression of CLEC3B may be an independent risk factor for disease‐free survival in lung cancer patients (HR = 0.655, 95% CI 0.430–0.996, Cox p = 0.048). In addition, we also investigated the potential role of CLEC3B in tumor-immune interactions and found that CLEC3B might be associated with the immune infiltration and immune activation of lung cancer, especially in squamous cell carcinoma.

**Conclusions:**

Our findings indicate that CLEC3B expression is downregulated in lung cancer and reveal the diagnostic and prognostic potential of CLEC3B in lung cancer and its potential as an immune-related therapeutic target in lung cancer.

## Background

Lung cancer, including both small-cell and non-small-cell types, is the second most common cancer in both men and women and by far the leading cause of cancer-related death worldwide [1]. Due to the high percentage of lung cancer patients diagnosed at locally advanced or extensive metastatic stages, the prognosis in the majority of patients at the time of diagnosis is still poor [2, 3]. Lung cancer patients diagnosed in an early stage and treated by surgery and radiotherapy showed better outcomes. However, with current diagnostic methods, such as computed tomography and positron emission tomography, there are still approximately 40% of lung cancer patients are diagnosed with distant metastasis [4]. Therefore, identifying effective biomarkers for early diagnosis and prognostication is important for reducing the mortality rate of lung cancer patients.

Increasing evidence has shown that the interaction between cancer cells and the tumor microenvironment, specifically the immune microenvironment, is also believed to be a key factor and showed to be involved in the tumor progression and therapy [5]. In recent years, T cell checkpoint inhibitors have greatly improved therapeutic efficacy in multiple cancers. Inhibitors of programmed cell death receptor-1 (PD-1) and its ligand (PD-L1) have shown promising antitumor effects in various cancers, including non-small-cell lung cancer (NSCLC) [6]. In addition, there is accumulating evidence that tumor-infiltrating immune cells affect the prognosis and efficacy of chemotherapy and immunotherapy [7]. Therefore, elucidating the immunophenotypes of tumor-immune interactions and identifying novel immune-related therapeutic targets in lung cancer are of particular importance.

C-type lectin domain family 3 member B (CLEC3B) encodes tetranectin, a plasminogen kringle-4-binding protein that is located in cell plasma, extracellular matrix and exosomes [8, 9]. Tetranectin plays a role in extracellular proteolysis by inducing plasminogen activation, which is associated with tumor invasion and metastasis [8, 10–13]. Moreover, CLEC3B has been reported in multiple cancers, including hepatocellular carcinoma, ovarian cancer, and oral squamous cell carcinoma, but a large number of in-depth studies are still needed to elucidate the molecular mechanism and specific function of CLEC3B in cancer progression [8, 14–19]. So far, research on CLEC3B has been very limited. Dai W. et al. reported that downregulation of exosomal CLEC3B in hepatocellular carcinoma promotes metastasis and angiogenesis via AMP-activated protein kinase and vascular endothelial growth factor signals [8]. Liu J et al. reported that CLEC3B has an anti-proliferation function mediated by the mitogen-activated protein kinase pathway in clear cell renal cell carcinoma [15]. To our knowledge, the relationship between CLEC3B and tumor immunity and its function in lung cancer has not been reported. We hypothesize that CLEC3B may serve as a potential diagnostic and prognostic biomarker and novel immune-related therapeutic target for lung cancer.

In this study, we used publicly available cancer databases to evaluate the prognostic and predictive role of CLEC3B expression, and to determine its correlation with the immune microenvironment phenotype.

## Materials and methods

In this study, we analyzed both SCLC and NSCLC.

### Publicly-available databases analysis

The Oncomine database was accessed to analyze CLEC3B mRNA expression in lung cancer. In this study, a *p* value of 0.0001, fold change of 2 and top 10% gene rank was set as the threshold. Eleven Oncomine datasets containing 1205 lung cancer samples were chosen to analyze the expression of CLEC3B in cancer vs noncancer tissues (Additional file 1: Table S1). Several datasets were obtained from the Gene Expression Omnibus (GEO) database to analyze the expression of CLEC3B between lung cancer and noncancer tissues. Gene expression profiles for adenocarcinoma (ADC) and squamous cell cancer (SCC) patients were obtained from the TCGA database (https://portal.gdc.cancer.gov/).

Estimation of Stromal and Immune cells in MAlignant Tumor tissues using Expression data (ESTIMATE) is a method that uses gene expression signatures to infer the fraction of stromal and immune cells in tumor samples [20]. Based on the gene expression profiles downloaded from the TCGA database, immune scores were calculated by the ESTIMATE algorithm using the R-package “estimate”. According to the rank of immune scores, we divided the ADC and SCC cases into two groups by the median value. The group with higher immune scores was defined as the high score group, and the other group was defined as the low score group.

Tumor immune estimation resource (TIMER) is a web server for comprehensive analysis of tumor-infiltrating immune cells (https://cistrome.shinyapps.io/timer/) [21]. The abundances of six immune infiltrates (B cells, CD4+ T cells, CD8+ T cells, neutrophils, macrophages and dendritic cells) can be estimated from gene expression profiles by a statistical method, which is validated using pathological estimations. In addition, the “correlation” module can create scatterplots illustrating the expression of a pair of genes in a particular cancer type and also generates the Spearman correlation and estimated statistical significance, which can be adjusted by tumor purity (the proportion of cancer cells in the admixture) or age. We used this module to explore the correlations between CLEC3B expression and gene markers of immune infiltrating cells in ADC and SCC.

### cDNA chip and real-time PCR

The cDNA chip (cDNA-HLugC030PT01, Xinchao, Shanghai, China) comprised 15 pairs of lung tumor and adjacent nontumor samples from 8 ADC patients and 7 SCC patients (Additional file 2: Table S2). The mRNA expression of CLEC3B was detected by real-time PCR, and β-actin acted as the internal control gene. PCR amplification was performed with ChamQ SYBR qPCR Master Mix (Q311-02, Vazyme, Nanjing, China) using the QuantStudio 6 Flex Real-Time PCR System (Thermo Fisher Scientific, Waltham, MA, USA) and the PCR parameters was provided in Additional file 3: the supplementary methods. The expression fold-changes were analyzed by the 2^−ΔΔCt^ relative quantitative methods. The real-time PCR primer sequences were as follows:

β-actin forward: 5′-GAAGAGCTACGAGCTGCCTGA-3′, reverse: 5′-CAGACAGCACTGTGTTGGCG-3′; CLEC3B forward: 5′-CCCAGACGAAGACCTTCCAC-3′, reverse: 5′-CGCAGGTACTCATACAGGGC-3′.

### Lung cancer tissue microarray

A lung cancer tissue microarray LAC-1403 was purchased from Servicebio Technology Co (Wuhan, China), including 34 pairs of lung tumor and peritumor tissues. The clinicopathologic characteristics of the patients are listed in Additional file 4: Table S3. The protein expression of CLEC3B was detected by immunohistochemistry, and the rabbit monoclonal anti-tetranectin (encoded by CLEC3B) antibody was purchased from Abcam (Cambridge, UK; ab108999). The protocol of immunohistochemistry was provided in Additional file 3: the supplementary methods. The histochemistry score (H-SCORE) was calculated out to assess the immunohistochemical results. H-SCORE = (percentage of cells of weak intensity × 1) + (percentage of cells of moderate intensity × 2) + (percentage of cells of strong intensity × 3). The percentage of stained area and intensity of stained cells were scored as 0, negative; 1+, weak; 2+, moderate; 3+, strong.

### Gene set enrichment analysis

Gene set enrichment analysis (GSEA) is a computational method that determines whether a predefined set of genes has statistically significant, concordant differences between two biological states [22]. TCGA datasets of ADC and SCC with a functional gene set file (c5.all.v5.1) gene set were analyzed by GSEA to obtain biological processes enriched by CLEC3B. The samples were divided into a high CLEC3B expression group (top 50%) and a low CLEC3B expression group (bottom 50%). Gene sets with nominal p‑value < 0.05 and FDR < 0.25 were considered statistically significant.

### Statistical analysis

SPSS version (v. 21.0) and GraphPad Prism (v. 8.0) were used for statistical analysis and generating figures. Paired t‑test, unpaired t-test and one‐way ANOVA followed by Dunnett’s test were used to compare the expression of CLEC3B in different groups. A Chi-square test was performed to analyze the correlation of CLEC3B expression and clinicopathological factors. The diagnostic value of CLEC3B in lung cancer was revealed by receiver operating characteristic (ROC) curves. The Kaplan–Meier Plotter (http://www.kmplot.com) was used to evaluate the prognostic value of CLEC3B in lung cancer [23]. According to the automatically selected best cutoff of CLEC3B expression, the patient samples were divided into a high expression group and a low expression group. The overall survival (OS) and progression-free survival (PFS) of lung cancer patients were evaluated, together with the log‑rank P‑values and hazard ratio (HR) with 95% confidence intervals (CIs). Kaplan–Meier survival analysis of the GEO database (GSE30219 and GSE31210) was used to investigate the prognostic significance of CLEC3B, and the log-rank P-value was calculated. A Cox proportional hazards regression model was applied for the univariate and multivariate analyses of survival. The factors with prognostic significance in the univariate analysis were included in the subsequent multivariate analysis. p-values < 0.05 were considered statistically significant.

## Results

### CLEC3B is downregulated in lung cancer and closely correlated with clinicopathological features

**Fig. 1:**
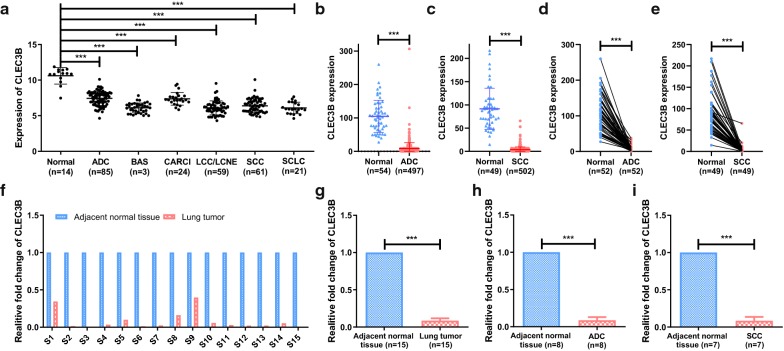
The expression of CLEC3B was downregulated in lung cancer. **a** CLEC3B expression in normal lung tissue and different histological subtypes of lung cancer in GSE30219. **b**, **c** CLEC3B expression in ADC and SCC vs normal lung in the TCGA dataset. **d**, **e** Expression levels of CLEC3B in paired ADC and SCC samples in the TCGA dataset. **f**–**i** Analysis of CLEC3B expression in 15 paired lung cancer specimens through real-time PCR. ADC, adenocarcinoma; BAS, basaloid; CARCI, carcinoid tumor; LCC, large-cell carcinoma; LCNE, large-cell neuroendocrine tumor; SCC, squamous cell carcinoma; SCLC, small-cell lung cancer. ***p < 0.001

To examine the expression of CLEC3B in lung cancer, we assessed the published data for cancer and normal tissues from the GEO and TCGA databases. The results showed a significant downregulation of CLEC3B in several histological subtypes (p < 0.001), including ADC, SCC, large-cell carcinoma (LCC), large-cell neuroendocrine tumor (LCNE) and small-cell lung cancer (SCLC) compared with noncancerous lung tissues in GSE30219 (Fig. 1a). Furthermore, CLEC3B was uniformly downregulated in ADC, SCC, LCC of GSE19188 (Additional file 5: Figure S1a) and 17 analyses of Oncomine (Additional file 5: Figure S1b). Consistently, comparison of CLEC3B gene expression across the RNA-seq data from TCGA demonstrated the downregulation of CLEC3B in both ADC and SCC (Fig. 1b, c). Data from cancer and matched adjacent nontumor tissues of ADC and SCC patients in the TCGA further corroborated these results (Fig. 1d, e).

**Fig. 2:**
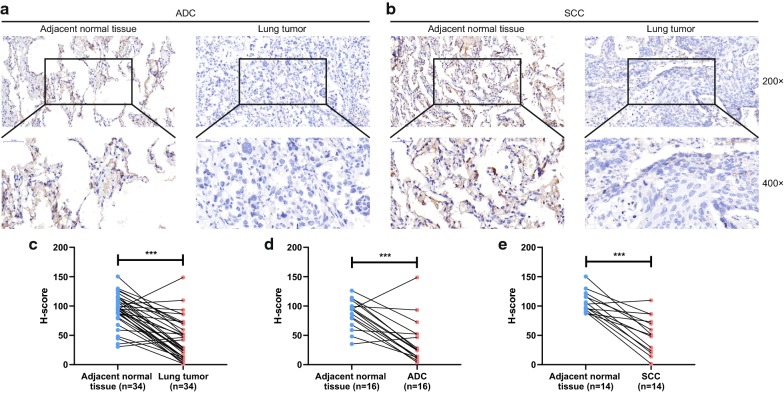
Immunohistochemistry detection of CLEC3B in lung cancer and normal tissues. **a**, **b** Representative images of immunohistochemistry staining (magnification, ×200, ×400) of ADC and SCC patients (the H-scores were 3.916 and 78.929 for image A; 20.068 and 106.444 for image B). **c**–**e** H-scores of clinical tumor and peritumor tissues for lung cancer samples. ADC, adenocarcinoma; SCC, squamous cell carcinoma; H-score, histochemistry score. ***p < 0.001

To further validate these results, the cDNA chip of lung cancer clinical samples was purchased and real-time PCR was carried out to assess CLEC3B mRNA expression. As expected, in comparison with matched adjacent nontumor tissues, a significant downregulation of CLEC3B was revealed in lung cancer tissues (Fig. 1f, g) and CLEC3B expression was downregulated in both ADC and SCC samples (Fig. 1h, i). In addition, the protein expression of CLEC3B was investigated by IHC staining using a tissue microarray, which also showed that CLEC3B had lower expression in tumor tissues than peritumor tissues (Fig. 2a, b). A paired t-test of H-scores revealed that the downregulation of CLEC3B in lung cancer was significant (P < 0.001) (Fig. 2c). The difference in ADC and SCC H-score was also significant (Fig. 2d, e).

To identify the correlation between CLEC3B expression and clinicopathological features, GSE30219 and GSE31210 were analyzed. We found that CLEC3B downregulation was closely associated with T stage (p < 0.001), N stage (p < 0.001), pathological stage (p < 0.001), and smoking (p < 0.001) (Tables 1, 2).

**Table 1:** Correlation between CLEC3B expression and the clinicopathological features of lung cancer patients in GSE30219

Characteristics	No. of patients	CLEC3B expression		Chi square value	p-value
		Low	High		
Age (years)				0.434	0.510
≤ 55	79	42	37		
> 55	213	104	109		
Gender				7.742	0.005
Male	250	133	117		
Female	43	13	30		
T stage				45.647	0.000
1	166	58	108		
2	69	40	29		
3	31	25	6		
4	21	20	1		
N stage				44.990	0.000
0	198	72	126		
1	53	42	11		
2–3	40	31	9		
M stage				0.514	0.473
0	282	140	142		
1	8	5	3

**Table 2:** Correlation between CLEC3B expression and the clinicopathological features of lung cancer patients in GSE31210

Characteristics	No. of patients	CLEC3B expression		Chi square value	p-value
		Low	High		
Age (years)				0.091	0.763
≤ 55	60	31	29		
> 55	166	82	84		
Gender				3.006	0.083
Male	121	54	67		
Female	105	59	46		
Smoke				12.907	0.000
Non	115	44	71		
Yes	111	69	42		
Pathological stage				18.430	0.000
IA	114	46	68		
IB	54	24	30		
II	58	43	15		
P stage i or ii				18.184	0.000
I	168	70	98		
II	58	43	15		

### CLEC3B has high diagnostic value in lung cancer

**Fig. 3:**
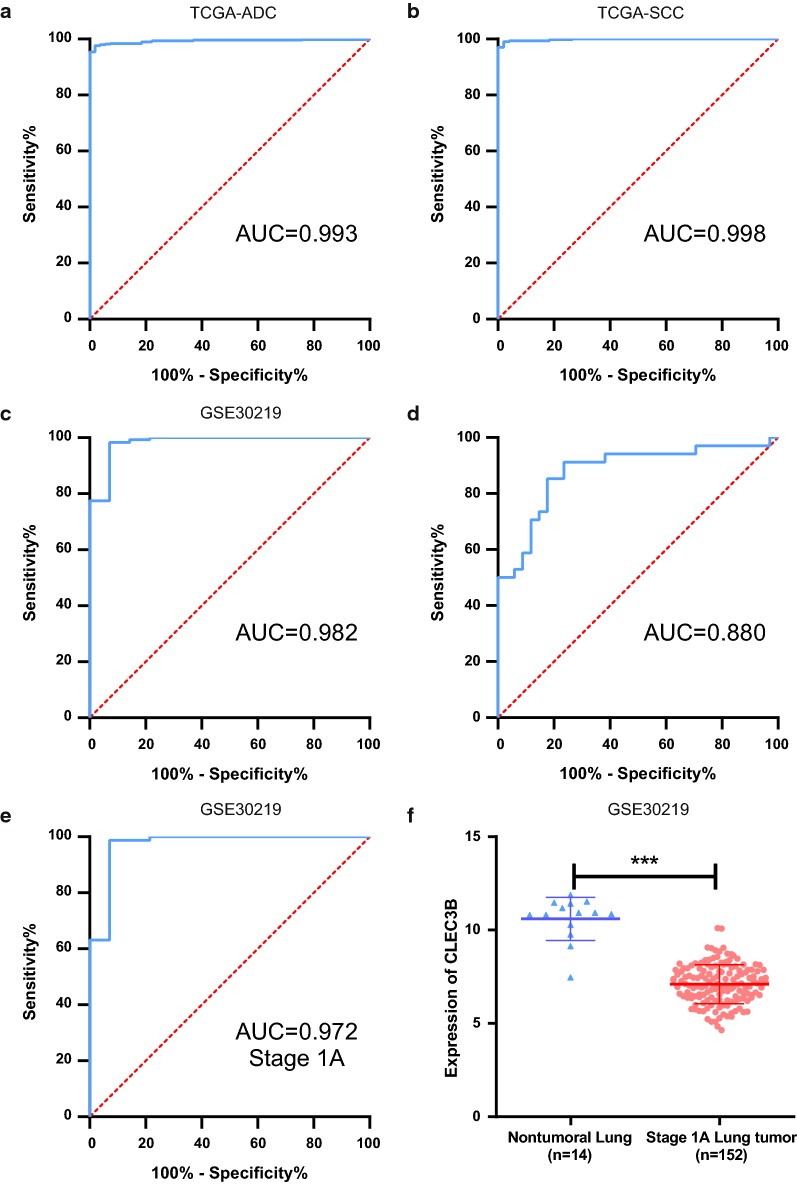
Diagnostic value of CLEC3B in lung cancer. **a**, **b** ROC curves for ADC patients and SCC patients in TCGA datasets. **c** ROC curve for all lung cancer patients in GSE30219. **d** ROC curve for lung cancer patients of tissue microarray LAC-1403. **e** ROC curve for stage IA lung cancer patients in GSE30219. **f** CLEC3B expression in normal lung and stage IA lung cancer in GSE30219. ROC curve, receiver operating characteristic curve; AUC, area under the receiver operating characteristic curve; ADC, adenocarcinoma; SCC, squamous cell carcinoma. ***p < 0.001

The GEO and TCGA datasets were used to evaluate the diagnostic potential of CLEC3B in lung cancer. The ROC analysis of TCGA-ADC revealed significant diagnostic accuracy with AUC = 0.993 (95% CI 0.987–0.999), sensitivity = 97.6% (95% CI 95.8–98.6%), and specificity = 98.2% (95% CI 90.2–99.9%) (Fig. 3a); the analysis of TCGA-SCC also showed accuracy with AUC = 0.998 (95% CI 0.996–1.00), sensitivity = 97.0% (95% CI 95.1–98.2%), and specificity = 100% (95% CI 92.7–100%) (Fig. 3b). As shown in Fig. 3c, CLEC3B also showed high diagnostic accuracy in GSE30219 with AUC = 0.982 (95% CI 0.952–1.00), sensitivity = 98.3% (95% CI 96.1–99.3%), and specificity = 92.9% (95% CI 68.5–99.6%). In addition, based on the clinical tissue samples H-SCORE mentioned above, the AUC value for the ability of CLEC3B to distinguish lung cancer tissue from nontumor tissue was 0.880 (95% CI 0.795–0.964), with a sensitivity of 91.2% (95% CI 77.0–96.9%) and a specificity of 76.5% (95% CI 60.0–87.6%) (Fig. 3d). Furthermore, we separated the stage IA patients and analyzed the diagnostic value of CLEC3B in GSE30219 with results of AUC = 0.972 (95% CI 0.922–1.00), sensitivity = 98.7% (95% CI 95.3–99.8%), and specificity = 92.9% (95% CI 68.5–99.6%) (Fig. 3e). CLEC3B was significantly downregulated in stage IA lung cancer patients (Fig. 3f). These results revealed that CLEC3B had a high diagnostic performance in differentiating lung cancer patients from normal individuals, even for the early stages of lung cancer.

### Decreased CLEC3B expression is a predictor of poor prognosis in lung cancer

**Fig. 4:**
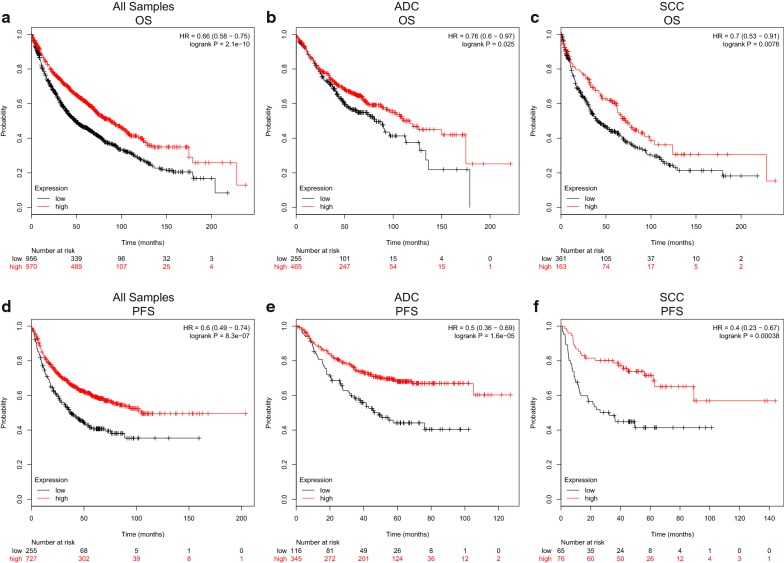
Kaplan–Meier survival curves comparing lung cancer patients with high and low expression of CLEC3B. **a** OS survival curves of all lung cancer patients (n = 1926), **b** ADC patients (n = 720), and **c** SCC patients (n = 524). **d**–**f** PFS survival curves of all lung cancer patients (n = 982), ADC patients (n = 461), and SCC patients (n = 141). OS, overall survival; PFS, progression-free survival; HR, hazard ratio; CI, confidence interval; ADC, adenocarcinoma; SCC, squamous cell carcinoma

Using the Kaplan‐Meier Plotter database and GEO datasets, we investigated the prognostic value of CLEC3B. Patients were split according to the automatically selected best cutoff value of CLEC3B expression. The Kaplan–Meier curve and log-rank test analyses revealed that among all patients, those with lower expression of CLEC3B had significantly poorer OS than those with high expression (HR = 0.66, 95% CI 0.58–0.75, p = 2.1e−10) (Fig. 4a); this was also true for ADC patients (HR = 0.76, 95% CI 0.60–0.97, p = 0.025) (Fig. 4b) and SCC patients (HR = 0.70, 95% CI 0.53–0.91, p = 0.0076) (Fig. 4c). In addition, the results showed that low expression of CLEC3B predicted poorer PFS than high expression in all lung cancer patients (HR = 0.60, 95% CI 0.49–0.74, p = 8.3e−07), ADC patients (HR = 0.50, 95% CI 0.36–0.69, p = 1.6e−05) and SCC patients (HR = 0.40, 95% CI 0.23–0.67, p = 0.00038) (Fig. 4d–f).

To better understand the effect of low expression of CLEC3B on survival, the Kaplan–Meier plotter database was accessed to analyze the correlation between CLEC3B expression and clinical characteristics of patients. Downregulation of CLEC3B was linked to worse OS and PFS in stage 1 to 2, stage T1 to T2 and stage N0 to N1 lung cancer patients (p < 0.05). In addition, the low expression of CLEC3B predicted poor OS and PFS in both smoking and nonsmoking patients (p < 0.05) (Table 3). Cox regressive models were performed to further evaluate the prognostic potential of CLEC3B expression. CLEC3B expression (HR = 0.565, 95% CI 0.423–0.753, Cox p = 0.000) was a significant predictor of OS in the univariate analysis (Table 4). The multivariate analysis value for disease‐free survival (DFS) was significant (HR = 0.655, 95% CI 0.430–0.996, Cox p = 0.048) but not for OS (Table 5). Therefore, the lower expression of CLEC3B may be defined as an independent risk factor for DFS in lung cancer patients. Furthermore, low expression of CLEC3B, which is capable of predicting poor OS and DFS, was also validated in the GSE30219 (Additional file 6: Figure S2 a, b) and GSE30210 datasets (Additional file 6: Figure S2 c, d).

**Table 3:** Correlation of CLEC3B expression and prognosis in lung cancer with clinicopathological factors by Kaplan-Meier plotter

Clinicopathological characteristics	Overall survival (n = 1928)			Progression-free survival (n = 982)		
	*N*	HR (95% CI)	p-value	*N*	HR (95% CI)	p-value
Sex						
Female	715	0.64 (0.5–0.81)	*0.00022*	468	0.59 (0.44–0.80)	*0.00051*
Male	1100	0.69 (0.59–0.81)	*5.9e−06*	514	0.62 (0.48–0.80)	*0.00026*
Stage						
1	577	0.58 (0.44–0.76)	*5.85e−05*	325	0.51 (0.33–0.81)	*0.0033*
2	244	1.61 (1.06–2.45)	*0.024*	130	0.40 (0.20–0.81)	*0.0089*
3	70	1.49 (0.83–2.70)	0.18	19	na	na
4	4	na	na	0	na	na
T stage						
1	437	0.64 (0.48–0.86)	*0.0024*	177	0.49 (0.29–0.84)	*0.008*
2	589	0.66 (0.52–0.83)	*0.00044*	351	0.50 (0.34–0.74)	*4e−04*
3	81	1.69 (1.03–2.78)	*0.037*	21	0.65 (0.24–1.80)	0.41
4	46	0.50 (0.26–0.96)	*0.033*	7	na	na
N stage						
0	781	0.69 (0.56–0.86)	*0.00066*	374	0.59 (0.43–0.82)	*0.0013*
1	252	0.67 (0.48–0.92)	*0.014*	130	0.38 (0.21–0.67)	*0.00051*
2	111	0.72 (0.45–1.14)	0.15	51	0.50 (0.25–1.00)	*0.047*
M stage						
0	681	0.61 (0.49–0.75)	*2.3e−06*	195	0.56 (0.33–0.94)	0.026
1	10	na	na	0	na	na
Grade						
I	201	0.84 (0.58–1.21)	0.35	140	0.68 (0.42–1.10)	0.12
II	310	0.63 (0.45–0.87)	*0.0052*	165	0.48 (0.30–0.77)	*0.002*
III	77	0.47 (0.24–0.91)	*0.022*	51	1.47 (0.64–3.35)	0.36
Smoke						
Ever	820	0.76 (0.62–0.93)	*0.0082*	603	0.58 (0.44–0.75)	*2.7e−05*
Never	205	0.41 (0.23–0.72)	*0.0013*	193	0.48 (0.29–0.79)	*0.0032*

Values in italics indicate p < 0.05

CI, confidence interval; HR, hazard ratio; na, not applicable

**Table 4:** Univariate and multivariate analyses for lung cancer patients on overall survival in the GSE30219

Variable	Univariate analysis		Multivariate analysis	
	HR (95% CI)	p-value	HR (95% CI)	p-value
Age (≤ 55 vs >5 5 years)	1.039 (1.025–1.054)	0.000	1.041 (1.026–1.056)	0.000
Gender				
Female vs male	0.573 (0.360–0.910)	0.018	0.670 (0.419–1.071)	0.094
T stage				
T1 vs T2 vs T3 vs T4	1.625 (1.415-1.866)	0.000	1.311 (1.082–1.590)	0.006
N stage				
N0 vs N1 vs N2 vs N4	1.730 (1.472–2.034)	0.000	1.371 (1.097–1.715)	0.006
M stage				
M0 vs M1	2.718 (1.114–6.631)	0.028	2.432 (0.987–5.997)	0.054
CLEC3B expression				
High vs low	0.565 (0.423–0.753)	0.000	0.789 (0.577–1.077)	0.136

CI, confidence interval; HR, hazard ratio

**Table 5:** Univariate and multivariate analyses for lung cancer patients on disease‐free survival in the GSE30219

Variable	Univariate analysis		Multivariate analysis	
	HR (95% CI)	p-value	HR (95% CI)	p-value
Age (≤ 55 vs > 55 years)	1.027 (1.009–1.045)	0.003	1.029 (1.010–1.048)	0.003
Gender				
Female vs male	0.746 (0.433-1.285)	0.290		
T stage				
T1 vs T2 vs T3 vs T4	1.882 (1.587–2.232)	0.000	1.309 (1.027–1.670)	0.030
N stage				
N0 vs N1 vs N2 vs N4	2.618 (1.791–2.624)	0.000	1.652 (1.259–2.169)	0.000
M stage				
M0 vs M1	3.837 (1.408–10.456)	0.009	3.951 (1.428–10.933)	0.008
CLEC3B expression				
High vs low	0.442 (0.300–0.650)	0.000	0.655 (0.430–0.996)	0.048

CI, confidence interval; HR, hazard ratio

### CLEC3B expression is associated with immune infiltration in lung cancer

Cancer patients diagnosed with the same histology types may have different immune infiltration levels, which could lead to diverse clinical outcomes [21, 24]. The increased number of tumor-infiltrating lymphocytes in primary tumor tissue relating to good prognosis has been reported in several cancers, including NSCLC [25, 26]. Therefore, the correlation of CLEC3B and immune infiltration levels was evaluated to reveal the possible mechanism by which CLEC3B affects the prognosis of lung cancer.

**Fig. 5:**
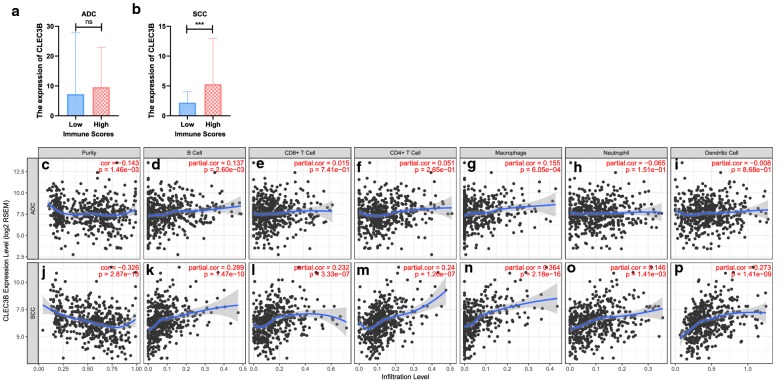
Correlation of CLEC3B expression with immune infiltration level in ADC and SCC. **a**, **b** Comparison of CLEC3B expression between the high and low immune score groups of ADC and SCC. **c**–**p** CLEC3B is related to tumor purity and immune infiltration levels of ADC and SCC by TIMER analysis. ADC, adenocarcinoma; SCC, squamous cell carcinoma; ns, no significance. ***p < 0.001

The immune scores of the patient samples were calculated by the ESTIMATE algorithm using the data from TCGA database to predict the presence of infiltrating immune cells in tumor tissues. According to the rank of immune scores, we divided the ADC and SCC cases into high and low score groups by the median value. The results showed that CLEC3B was upregulated in the high immune score group of SCC, but there was no statistical significance in ADC, which indicated that CLEC3B might be involved in the immune infiltration of SCC (Fig. 5a, b).

To further validate the relationship between CLEC3B expression and immune infiltration of lung cancer, we used TIMER to evaluate the correlations of CLEC3B expression with tumor purity and infiltrating levels of immune cells. The results showed that CLEC3B expression was slightly correlated with ADC tumor purity but had a more significant correlation with tumor purity and immune infiltration levels of SCC. In ADC, the expression level of CLEC3B had no significant correlations with the infiltration of CD8+ T cells, CD4+ T cells, neutrophils and dendritic cells (DCs) (Fig. 5c-i). However, in SCC, the expression of CLEC3B was significantly positively correlated with the infiltration of B cells (r = 0.289, p = 1.47e−10), CD8+ T cells (r = 0.232, p = 3.33e−07), CD4 + T cells (r = 0.24, p = 1.20e−07), macrophages (r = 0.364, p = 2.18e−16), neutrophils (r = 0.146, p = 1.41e−03) and DCs (r = 0.273, p = 1.41e−09) (Fig. 5j–p). These findings suggest that CLEC3B may play an important role in immune infiltration of SCC.

The correlations between CLEC3B and immune markers of immune cells (selected by investigating some relevant papers [27–31]) were analyzed in the TIMER database to explore the relationship between CLEC3B and immune infiltrating cells of ADC and SCC (Table 6). In SCC, CLEC3B expression was positively correlated with majority of gene markers of different functional T cells (CD8+ T, Th1, Th2, etc.), B cells, dendritic cells, neutrophils and natural killer cells after adjusting for purity, which was consistent with the results in Fig. 5. Moreover, CLEC3B was related to tumor-associated macrophage (TAM) infiltration, which exerts both anti- and pro-tumor effects. However, CLEC3B expression only had a strong correlation with few markers for B cells, DCs, neutrophils, natural killer cells and Th1 cells in ADC. The results showed that the expression level of CLEC3B was significantly correlated with most immune markers of immune cells in SCC but only a few markers in ADC. These findings may provide an explanation for the difference in the prognostic value of CLEC3B in ADC and SCC.

**Table 6:** Correlation analysis between CLEC3B and relate genes and markers of immune cells in TIMER

Description	Gene markers	ADC				SCC			
		None		Purity		None		Purity	
		Cor	P	Cor	P	Cor	P	Cor	P
CD8+ T cell	CD8A	0.07	0.113	0.003	0.95	0.322	***	0.238	***
	CD8B	0.08	0.071	0.035	0.438	0.276	***	0.232	***
	CD45	0.2	***	0.126	**	0.447	***	0.361	***
T cell (general)	CD3D	0.138	*	0.066	0.144	0.382	***	0.279	***
	CD3E	0.168	**	0.101	0.024	0.397	***	0.294	***
	CD2	0.155	**	0.083	0.067	0.382	***	0.282	***
B cell	CD19	0.194	***	0.145	*	0.414	***	0.316	***
	CD79A	0.154	**	0.105	0.020	0.385	***	0.279	***
	CD27	0.148	***	0.091	*	0.384	***	0.289	***
	CD20	0.293	***	0.249	***	0.481	***	0.415	***
Monocyte	CD14	0.065	0.139	0	0.994	0.391	***	0.281	***
	CD115 (CSF1R)	0.11	0.012	0.04	0.373	0.449	***	0.357	***
TAM	CCL2	0.03	0.5	− 0.036	0.419	0.31	***	0.229	***
	CD68	0.165	**	0.11	0.015	0.372	***	0.27	***
	IL10	0.198	***	0.132	*	0.41	***	0.335	***
M1 Macrophage	INOS (NOS2)	0.236	***	0.23	***	0.11	0.014	0.116	0.011
	CD80	0.101	*	0.021	0.643	0.302	***	0.217	***
	IRF5	0.037	0.4	− 0.021	0.635	0.152	**	0.116	0.011
	IL6	0.02	0.659	− 0.02	0.658	0.203	***	0.154	***
	CD64 (FCGR1A)	0.012	0.792	− 0.069	0.127	0.404	***	0.316	***
M2 Macrophage	CD163	0.098	0.026	0.028	0.529	0.46	***	0.373	***
	CD206	0.306	***	0.256	***	0.456	***	0.374	***
	VSIG4	0.162	**	0.102	0.023	0.478	***	0.4	***
	MS4A4A	0.233	***	0.175	***	0.49	***	0.409	***
Neutrophils	CD66b (CEACAM8)	0.3	***	0.29	***	0.268	***	0.255	***
	CD11b (ITGAM)	0.101	0.022	0.03	0.5	0.379	***	0.27	***
	CD15	0.017	0.706	0.001	0.976	0.146	**	0.123	**
Natural killer cell	KIR2DL1	0.213	***	0.203	***	0.174	***	0.128	***
	KIR2DL3	0.081	0.067	0.04	0.375	0.187	***	0.145	*
	KIR3DL1	0.142	*	0.123	*	0.272	***	0.221	***
	KIR3DL2	0.028	0.533	− 0.024	0.602	0.191	***	0.135	*
	CD56	0.208	***	0.21	***	0.211	***	0.253	***
	CD335 (NKp46)	0.024	0.592	− 0.018	0.694	0.237	***	0.183	***
Dendritic cell	BDCA-1 (CD1C)	0.415	***	0.38	***	0.524	***	0.446	***
	BDCA-3 (CD141)	0.4	***	0.363	***	0.519	***	0.45	***
	BDCA-4 (NRP1)	− 0.017	0.699	− 0.042	0.357	0.257	***	0.143	**
	CD123	0.347	***	0.308	***	0.535	***	0.468	***
	CD11c (ITGAX)	0.137	*	0.071	0.116	0.427	***	0.319	***
Th1	T-bet (TBX21)	0.134	*	0.064	0.159	0.365	***	0.269	***
	STAT4	0.138	*	0.078	0.084	0.431	***	0.344	***
	STAT1	− 0.204	***	− 0.276	***	0.026	0.56	-0.059	0.199
Th2	GATA3	− 0.033	0.457	− 0.114	0.012	0.203	***	0.12	*
	STAT6	0.242	***	0.239	***	0.123	*	0.111	0.016
	IL13	0.148	**	0.104	0.021	0.235	***	0.186	***
Tfh	BCL6	0.058	0.189	0.068	0.134	0.075	0.092	0.102	0.025
	IL21	− 0.049	0.267	− 0.085	0.061	0.196	***	0.122	*
Th17	STAT3	0.048	0.275	0.051	0.255	0.072	0.109	0.006	0.89
	IL17A	0.063	0.154	0.046	0.307	0.012	0.783	-0.051	0.266
	RORγt	0.202	***	0.242	***	0.517	***	0.469	***
Treg	FOXP3	0.003	0.947	− 0.081	0.072	0.281	***	0.162	**
	CD25	− 0.039	0.371	− 0.117	**	0.293	***	0.193	***
	CCR8	0.015	0.729	− 0.064	0.155	0.263	***	0.152	**
	STAT5B	0.196	***	0.18	***	0.112	0.012	0.095	0.037
T cell exhaustion	PD-1 (PDCD1)	0.01	0.816	− 0.065	0.149	0.346	**	0.249	***
	CTLA4	0.046	0.293	− 0.046	0.31	0.306	***	0.192	***
	LAG3	− 0.085	0.054	− 0.154	**	0.17	**	0.078	0.087
	TIM-3 (HAVCR2)	0.098	0.026	0.02	0.652	0.443	***	0.356	***
	GZMB	− 0.089	0.045	− 0.16	**	0.222	***	0.116	0.011

ADC, adenocarcinoma; SCC, squamous cell carcinoma; None, correlation without adjustment; Purity, correlation adjusted by purity; TAM, tumor-associated macrophage; Th, T helper cell; Tfh, Follicular helper T cell; Treg, regulatory T cell; Cor, R value of Spearman’s correlation

* P < 0.01; ** P < 0.001; *** P < 0.0001

### CLEC3B is involved in immune activation and proliferation inhibition in lung cancer

**Fig. 6:**
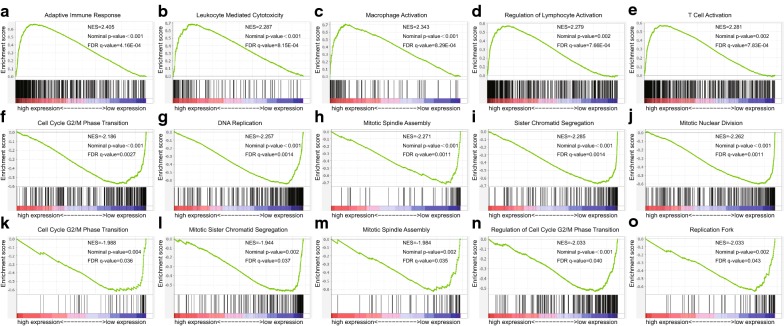
Immune activation and cell cycle processes were enriched according to CLEC3B expression in lung cancer. Gene set enrichment analysis was performed using the TCGA gene expression profiles of ADC and SCC, and the ʻc5.all.v5.1ʼ gene set was selected to process the analysis. **a**–**e** Immune activation processes were enriched in the CLEC3B high expression group of SCC. **f**–**j** Cell cycle processes were enriched in the CLEC3B low expression group of ADC. **k**–**o** Cell cycle processes were enriched in the CLEC3B low expression group of SCC; NES, normal enrichment score; FDR, false discovery rate

GSEA was used to explore the mechanisms of CLEC3B in lung cancer. The TCGA data of ADC and SCC were divided into high (top 50%) and low (bottom 50%) CLEC3B expression groups according to the median expression of CLEC3B. TCGA data were analyzed with GMT file C5 (GO gene set). The top 20 enrichment results (nominal p value < 0.05 and FDR < 0.25) are shown in Additional files 7, 8, 9, 10: Tables S4, S5, S6, S7. There were few enrichment results related to cancer progression in the CLEC3B high expression ADC, whereas in the CLEC3B high expression group of SCC, many gene sets related to immune activation were enriched, suggesting that CLEC3B may suppress the progression of SCC through immune activation, which is also consistent with the previous results (Fig. 6a–e). In addition, several cell cycle-related gene sets were enriched in the CLEC3B low expression group of ADC and SCC, which suggests that CLEC3B may also be involved in the inhibition of cell proliferation in lung cancer (Fig. 6f–o).

## Discussion

Lung cancer is the leading cause of cancer associated mortality. Cancer survival largely depends on the stage at diagnosis; however, 40–60% of lung cancer patients are not diagnosed until advanced stages [32]. In the present study, we observed a decrease in CLEC3B expression in lung cancer, which was associated with worse OS and DFS. Our results also suggested that CLEC3B is related to immune infiltration in lung cancer, which may be the mechanism by which CLEC3B affects prognosis. These results indicated that CLEC3B could serve as a potential biomarker for diagnosis and prognosis and could be considered as a novel immune-related therapeutic target.

CLEC3B, a member of the C-type lectin domain family, encodes the tetrameric protein tetranectin. Low expression of CLEC3B has been confirmed in several tumors. However, its expression level and function in lung cancer have not been reported previously. Database analysis and patient sample detection revealed that CLEC3B is significantly downregulated in various types of lung cancer. Furthermore, our results indicate that CLEC3B is significantly downregulated in stage IA and has great diagnostic value for lung cancer at an early stage, since the AUC value of CLEC3B for the diagnosis of stage IA lung cancer patients was approximately 1, which indicates great significance. Thus, our results revealed that the expression of CLEC3B can distinguish lung cancer patients from healthy individuals with high sensitivity and specificity.

We found that the downregulation of CLEC3B was linked to worse PFS and OS. In addition, univariate and multivariate analyses indicated that the lower expression of CLEC3B may be defined as a risk factor that affects the OS and DFS of lung cancer patients. As such, we validated that CLEC3B has prognostic value in lung cancer. Moreover, the CLEC3B expression level was negatively correlated with TNM stage. Patients with a low level of CLEC3B were more likely to present with disease in a late TNM stage, suggesting that CLEC3B is a tumor suppressor gene of lung cancer. In oral squamous cell carcinoma, CLEC3B has been reported as a tumor suppressor [33].

Recent years have witnessed the rapid development of tumor immunotherapy. There has been increasing recognition of the role of the immune system in cancer development and progression [34, 35]. The exploration of the tumor microenvironment as a prognostic and diagnostic biomarker or therapy target is an area of active research [36]. Some studies have shown that immune cell infiltration has an influence on survival in lung cancer [37, 38]. Other significant findings of the present study are that CLEC3B plays a role in tumor-immune interactions and that CLEC3B expression is correlated with the immune infiltration level in lung cancer, especially in SCC. Our results revealed a most significantly positive correlation between CLEC3B expression and the level of B cells, CD8+ T cells, CD4+ T cells, macrophages and DCs infiltration in SCC.

In the NSCLC tumor microenvironment, the landscape of immune cell types is T cells, followed by B cells, macrophages, DCs and natural killer cells [28]. Dendritic cells are specialized antigen-presenting cells that play an important role in the activation of antitumor T lymphocytes [39, 40]. The infiltration of T cells generally predicts a better clinical outcomes in patients [41]. In addition, the role of tumor-infiltrating B cells in the tumor microenvironment has attracted increasing attention. Most studies indicate that the infiltration of B cells in NSCLC is related to a favorable outcome [29]. In NSCLC patients who were not treated with PD-1/PD-L1 inhibitors, higher levels of T and B plasma cells were associated with better prognosis [42]. The results of GSEA further validated that CLEC3B may be involved in immune activation in SCC. In addition, our results showed that CLEC3B is related to macrophages in SCC. However, the role of macrophage infiltration and activation in lung cancer remains controversial [43]. Generally, TAMs play a pro-tumoral role; however, they could have the capacity to cooperate with T cells in anti-tumoral action under appropriate stimulation [44]. According to the latest report, high levels of M1, CD204 + M2, and macrophages infiltration are independent factors of favorable prognosis in stage I to III NSCLC patients [45]. Our results revealed that CLEC3B is capable of recruiting and regulating immune infiltrating cells in lung cancer. However, the precise role of CLEC3B in tumor immune microenvironment and tumor progression still needs further research exploration.

Taken together, our results indicate that CLEC3B may improve lung cancer patient prognosis through immune infiltration and immune activation. As to the different effects of CLEC3B on immune infiltration of SCC and ADC, we think this may be due to the differences in their intrinsic immune microenvironments [46]. It is worth pointing out that the prognostic value of CLEC3B in SCC was more significant than that in ADC (Fig. 4), which may be due to the stronger correlation between CLEC3B expression and immune infiltration in SCC than in ADC. In addition, we also found that CLEC3B may be related to the inhibition of cell proliferation in lung cancer, which is consistent with a report in clear cell renal cell carcinoma [15, 33]. The function of inhibiting cell proliferation is correlated with the prognosis of lung cancer patients [47]. Taken together, immune activation and proliferation inhibition induced by CLEC3B may be the two components of the gene’s anticancer effect.

## Conclusion

In conclusion, we found that CLEC3B is downregulated in lung cancer, and it may act as an early stage diagnostic marker in lung cancer patients. In addition, low expression of CLEC3B is associated with poor prognosis in lung cancer. Moreover, CLEC3B may promote tumor-induced immune response activation and immune infiltration in SCC and may inhibit the proliferation of lung cancer to play an anticancer role. Thus, we reported that CLEC3B is related to lung cancer and identified a possible potential diagnostic and prognostic biomarker and immune-related therapeutic target for lung cancer. Further studies are needed to confirm these results and reveal the underlying mechanisms.

## Supplementary information

**Additional file 1: Table S1.** Comparison of CLEC3B expression across 17 analyses.

**Additional file 2: Table S2.** Clinical information of 15 lung cancer samples of cDNA chip (cDNA-HLugC030PT01).

**Additional file 3.** Supplementary methods.

**Additional file 4: Table S3.** Clinical information of 34 lung cancer samples of tissue microarray (LAC-1403).

**Additional file 5: Figure S1.** Downregulation of CLEC3B in lung cancer. (a) Analysis of CLEC3B expression in normal lung and different histological subtypes of lung cancer in GSE19188. (b) Analysis of CLEC3B expression across 17 analyses of Oncomine. ADC, adenocarcinoma; SCC, squamous cell carcinoma; LCC, large-cell carcinoma. ***p < 0.001.

**Additional file 6: Figure S2.** Impact of CLEC3B expression on OS and DFS in lung cancer patients from the GEO datasets. (a, b) OS survival curves and DFS survival curves of lung cancer patients in GSE30219. (c, d) and in GSE31210. OS, overall survival; DFS, disease-free survival.

**Additional file 7: Table S4.** Enrichment of GO in the CLEC3B high expression group of ADC

**Additional file 8: Table S5.** Enrichment of GO in the CLEC3B low expression group of ADC.

**Additional file 9: Table S6.** Enrichment of GO in the CLEC3B high expression group of SCC.

**Additional file 10: Table S7.** Enrichment of GO in the CLEC3B low expression group of SCC.

## Data Availability

The datasets used and/or analyzed during the current study are available from the corresponding author upon reasonable request.

## Abbreviations

CLEC3B: C- type lectin domain family 3 member B
PD-1: Programmed cell death receptor-1
PD-L1: Programmed cell death ligand-1
NSCLC: Non-small-cell lung cancer
ADC: Adenocarcinoma
SCC: Squamous cell carcinoma
H-SCORE: Histochemistry score
TCGA: The Cancer Genome Atlas
ESTIMATE: Estimation of Stromal and Immune cells in MAlignant Tumor tissues using Expression data
OS: Overall survival
PFS: Progression-free survival
HR: Hazard ratio
CIs: Confidence intervals
TIMER: Tumor immune estimation resource
GSEA: Gene set enrichment analysis
ROC: Receiver operating characteristic curves
LCC: Large-cell carcinoma
LCNE: Large-cell neuroendocrine tumor
SCLC: Small-cell lung cancer
AUC: Area under the receiver operating characteristic curve
DFS: Disease‐free survival
