# Supplementary material for: CLEC3B as a potential diagnostic and prognostic biomarker in lung cancer and association with the immune microenvironment
Source: Cancer Cell Int. 2020 Apr 1;20:106. doi: 10.1186/s12935-020-01183-1 (PMC7110733; doi:10.1186/s12935-020-01183-1)
Supplement: Supplementary file 1 — Additional file 1: Table S1. Comparison of CLEC3B expression across 17 analyses. [file 12935_2020_1183_MOESM1_ESM.doc]

**Table S1 Comparison of CLEC3B expression across 17 analyses**

| **Legend** | **Dataset** | **PMID** | **No. of patients** | **17 analyses** | **P-value** | **Fold change** |
| --- | --- | --- | --- | --- | --- | --- |
| 1 | Beer et al,2002 | 12118244 | 96 | ADC vs normal | 7.44E-33 | -120.514 |
| 2 | Talbot et al,2005 | 15833835 | 93 | SCC vs normal | 9.19E-15 | -3.991 |
| 3 | Selamat et al,2012 | 22613842 | 116 | ADC vs normal | 6.00E-53 | -12.754 |
| 4 | Landi et al,2008 | 18297132 | 107 | ADC vs normal | 3.18E-37 | -10.634 |
| 5 | Wachi et al,2005 | 16188928 | 10 | SCC vs normal | 1.02E-06 | -11.932 |
| 6 | Su et al,2007 | 17540040 | 66 | ADC vs normal | 7.74E-17 | -17.8 |
| 7 | Bhattacharjee et al,2001 | 11707567 | 203 | SCC vs normal | 7.69E-10 | -19.787 |
| 8 |  |  |  | ADC vs normal | 1.10E-09 | -21.716 |
| 9 |  |  |  | LCT vs normal | 9.38E-11 | -28.647 |
| 10 |  |  |  | SCLC vs normal | 1.53E-05 | -41.862 |
| 11 | Stearman et al,2005 | 16314486 | 39 | ADC vs normal | 4.90E-11 | -9.41 |
| 12 | Garber et al,2001 | 11707590 | 73 | SCC vs normal | 6.30E-08 | -11.76 |
| 13 |  |  |  | ADC vs normal | 9.87E-06 | -5.006 |
| 14 | Hou et al,2010 | 20421987 | 156 | ADC vs normal | 1.18E-18 | -8.44 |
| 15 |  |  |  | LCC vs normal | 3.11E-13 | -20.159 |
| 16 |  |  |  | SCLC vs normal | 1.55E-18 | -16.099 |
| 17 | Okayama et al,2012 | 22080568 | 246 | ADC vs normal | 2.29E-17 | -7.675 |

**Abbreviations:** ADC, adenocarcinoma; LCT, lung carcinoid tumor; LCC, large cell carcinoma; SCC, squamous cell carcinoma; SCLC, small cell lung cancer.
