# Supplementary material for: CLEC3B as a potential diagnostic and prognostic biomarker in lung cancer and association with the immune microenvironment
Source: Cancer Cell Int. 2020 Apr 1;20:106. doi: 10.1186/s12935-020-01183-1 (PMC7110733; doi:10.1186/s12935-020-01183-1)
Supplement: Supplementary file 2 — Additional file 2: Table S2. Clinical information of 15 lung cancer samples of cDNA chip (cDNA-HLugC030PT01). [file 12935_2020_1183_MOESM2_ESM.docx]

**Table S2** Clinical information of 15 lung cancer samples of cDNA chip (cDNA-HLugC030PT01)

| **Sample no.** | **Gender** | **Age (years)** | **Pathological type** |
| --- | --- | --- | --- |
| S1 | F | 44 | Adenocarcinoma |
| S2 | M | 66 | Adenocarcinoma |
| S3 | M | 74 | Adenocarcinoma |
| S4 | F | 64 | Adenocarcinoma |
| S5 | F | 63 | Adenocarcinoma |
| S6 | M | 68 | Adenocarcinoma |
| S7 | M | 74 | Adenocarcinoma |
| S8 | F | 47 | Adenocarcinoma |
| S9 | M | 64 | Squamous carcinoma |
| S10 | M | 63 | Squamous carcinoma |
| S11 | M | 74 | Squamous carcinoma |
| S12 | M | 65 | Squamous carcinoma |
| S13 | M | 55 | Squamous carcinoma |
| S14 | M | 75 | Squamous carcinoma |
| S15 | M | 56 | Squamous carcinoma |
