# Supplementary material for: CLEC3B as a potential diagnostic and prognostic biomarker in lung cancer and association with the immune microenvironment
Source: Cancer Cell Int. 2020 Apr 1;20:106. doi: 10.1186/s12935-020-01183-1 (PMC7110733; doi:10.1186/s12935-020-01183-1)
Supplement: Supplementary file 3 — Additional file 3. Supplementary methods. [file 12935_2020_1183_MOESM3_ESM.doc]

**Real-time PCR parameters**

PCR amplification was performed with the ChamQ SYBR qPCR Master Mix (Q311-02, Vazyme, Nanjing, China) using the QuantStudio 6 Flex Real-Time PCR System (Thermo Fisher Scientific, Waltham, MA, USA). Parameters of real-time PCR were: 2min 50°C, 10 min 95°C, and then 15s 95°C, 1 min 60°C for 40 cycles.

**Immunohistochemistry**

Paraffin-embedded tissues sections were deparaffinized with a histologic clearing agent and a series of rehydration ethanol washes. Antigen retrieval was performed by boiling samples in 10 mm sodium citrate buffer, pH 6, with 0.05% Tween 20 in a microwave for 20 min. To block endogenous peroxidase, each slide was incubated with 3% hydrogen peroxide for 30 min and then 10% normal goat serum was used to block. Sections were incubated overnight at 4°C with a specific primary antibody for tetranectin (encoded by CLEC3B) (ab108999; Abcam, Cambridge, UK). Slides were rinsed with PBST and incubated for 1 h at room temperature with secondary antibody. Staining was visualized with 3, 3-diaminobenzidine, and were counterstained with hematoxylin.
