# Supplementary material for: CLEC3B as a potential diagnostic and prognostic biomarker in lung cancer and association with the immune microenvironment
Source: Cancer Cell Int. 2020 Apr 1;20:106. doi: 10.1186/s12935-020-01183-1 (PMC7110733; doi:10.1186/s12935-020-01183-1)
Supplement: Supplementary file 4 — Additional file 4: Table S3. Clinical information of 34 lung cancer samples of tissue microarray (LAC-1403). [file 12935_2020_1183_MOESM4_ESM.docx]

**Table S3** Clinical information of 34 lung cancer samples of tissue microarray (LAC-1403)

| **Sample no.** | **Gender** | **Age (years)** | **Pathological type** |
| --- | --- | --- | --- |
| S1 | F | 46 | ADC |
| S2 | M | 74 | ADC |
| S3 | M | 59 | ADC |
| S4 | M | 67 | SCC |
| S5 | F | 62 | ADC |
| S6 | M | 68 | ADC |
| S7 | M | 52 | NEC |
| S8 | M | 61 | SCLC |
| S9 | F | 45 | ADC |
| S10 | M | 56 | SCC |
| S11 | M | 76 | SCC |
| S12 | F | 58 | SCC |
| S13 | F | 66 | ADC |
| S14 | M | 61 | ADC |
| S15 | M | 63 | ADC |
| S16 | M | 58 | ASC |
| S17 | M | 73 | SCC |
| S18 | M | 61 | SCC |
| S19 | M | 67 | SCC |
| S20 | M | 65 | NEC |
| S21 | F | 59 | ADC |
| S22 | F | 73 | ADC |
| S23 | F | 63 | SCC |
| S24 | M | 71 | SCC |
| S25 | M | 54 | SCC |
| S26 | M | 67 | SCC |
| S27 | M | 56 | SCC |
| S28 | F | 53 | ADC |
| S29 | M | 74 | SCC |
| S30 | M | 52 | ADC |
| S31 | M | 56 | SCC |
| S32 | M | 67 | ADC |
| S33 | F | 67 | ADC |
| S34 | F | 59 | ADC |

**Abbreviations:** ADC, adenocarcinoma; ASC, adenosquamous carcinoma.; NEC, neuroendocrine carcinoma; SCC, squamous cell carcinoma; SCLC, small cell lung cancer.
