# Supplementary material for: CLEC3B as a potential diagnostic and prognostic biomarker in lung cancer and association with the immune microenvironment
Source: Cancer Cell Int. 2020 Apr 1;20:106. doi: 10.1186/s12935-020-01183-1 (PMC7110733; doi:10.1186/s12935-020-01183-1)
Supplement: Supplementary file 5 — Additional file 5: Figure S1. Downregulation of CLEC3B in lung cancer. (a) Analysis of CLEC3B expression in normal lung and different histological subtypes of lung cancer in GSE19188. (b) Analysis of CLEC3B expression across 17 analyses of Oncomine. ADC, adenocarcinoma; SCC, squamous cell carcinoma; LCC, large-cell carcinoma. ***p < 0.001. [file 12935_2020_1183_MOESM5_ESM.pdf]

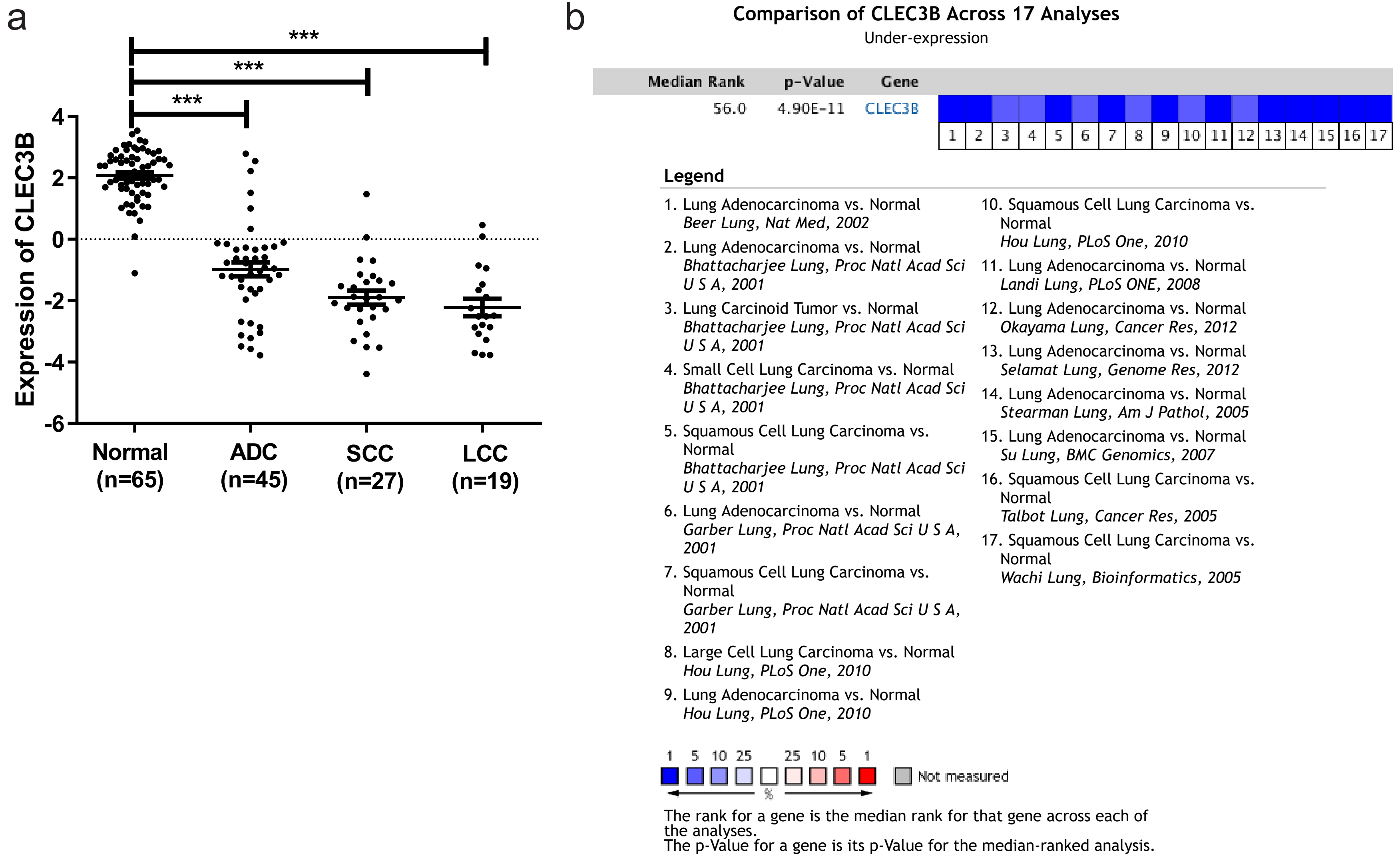
a
***
***
***
Expression of CLEC3B
4
2
0
-2
-4
-6
Normal (n=65)
ADC (n=45)
SCC (n=27)
LCC (n=19)
b
Comparison of CLEC3B Across 17 Analyses
Under-expression
Median Rank
p-Value
Gene
56.0
4.90E-11
CLEC3B
1 2 3 4 5 6 7 8 9 10 11 12 13 14 15 16 17
Legend
1. Lung Adenocarcinoma vs. Normal Beer Lung, Nat Med, 2002
2. Lung Adenocarcinoma vs. Normal Bhattacharjee Lung, Proc Natl Acad Sci U S A, 2001
3. Lung Carcinoid Tumor vs. Normal Bhattacharjee Lung, Proc Natl Acad Sci U S A, 2001
4. Small Cell Lung Carcinoma vs. Normal Bhattacharjee Lung, Proc Natl Acad Sci U S A, 2001
5. Squamous Cell Lung Carcinoma vs. Normal Bhattacharjee Lung, Proc Natl Acad Sci U S A, 2001
6. Lung Adenocarcinoma vs. Normal Garber Lung, Proc Natl Acad Sci U S A, 2001
7. Squamous Cell Lung Carcinoma vs. Normal Garber Lung, Proc Natl Acad Sci U S A, 2001
8. Large Cell Lung Carcinoma vs. Normal Hou Lung, PLoS One, 2010
9. Lung Adenocarcinoma vs. Normal Hou Lung, PLoS One, 2010
10. Squamous Cell Lung Carcinoma vs. Normal Hou Lung, PLoS One, 2010
11. Lung Adenocarcinoma vs. Normal Landi Lung, PLoS ONE, 2008
12. Lung Adenocarcinoma vs. Normal Okayama Lung, Cancer Res, 2012
13. Lung Adenocarcinoma vs. Normal Selamat Lung, Genome Res, 2012
14. Lung Adenocarcinoma vs. Normal Stearman Lung, Am J Pathol, 2005
15. Lung Adenocarcinoma vs. Normal Su Lung, BMC Genomics, 2007
16. Squamous Cell Lung Carcinoma vs. Normal Talbot Lung, Cancer Res, 2005
17. Squamous Cell Lung Carcinoma vs. Normal Wachi Lung, Bioinformatics, 2005
1 5 10 25 25 10 5 1
Not measured
%
The rank for a gene is the median rank for that gene across each of the analyses.
The p-Value for a gene is its p-Value for the median-ranked analysis.
