# Supplementary figures and images for: CLEC3B as a potential diagnostic and prognostic biomarker in lung cancer and association with the immune microenvironment
Source: Cancer Cell Int. 2020 Apr 1;20:106. doi: 10.1186/s12935-020-01183-1 (PMC7110733; doi:10.1186/s12935-020-01183-1)

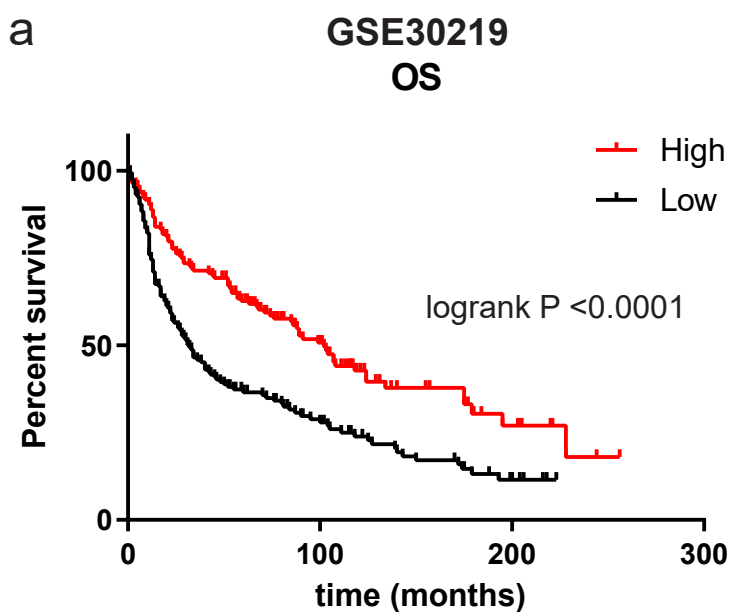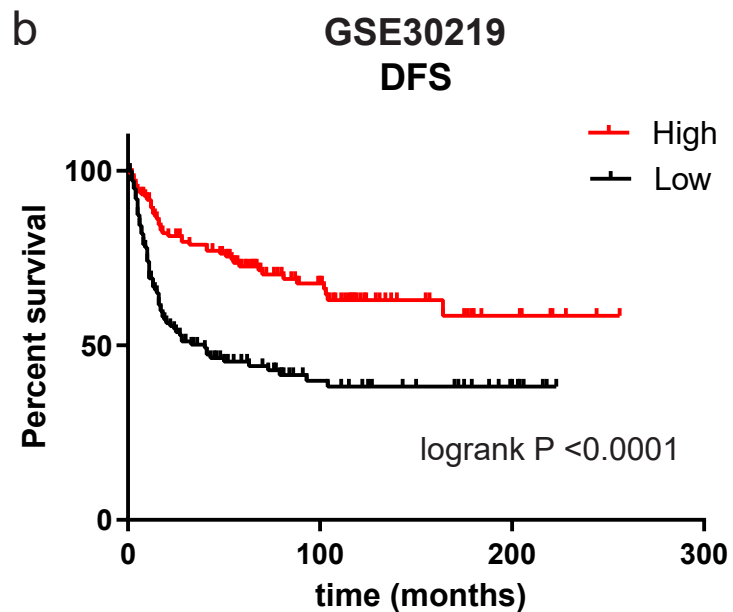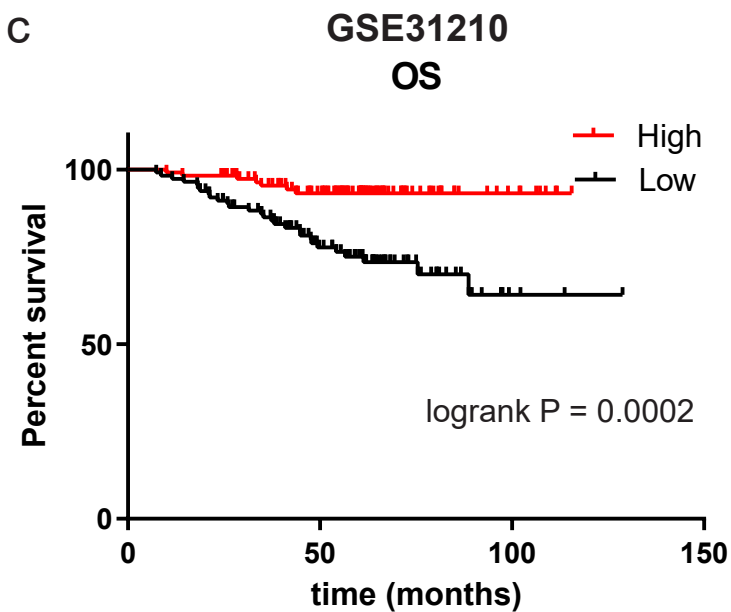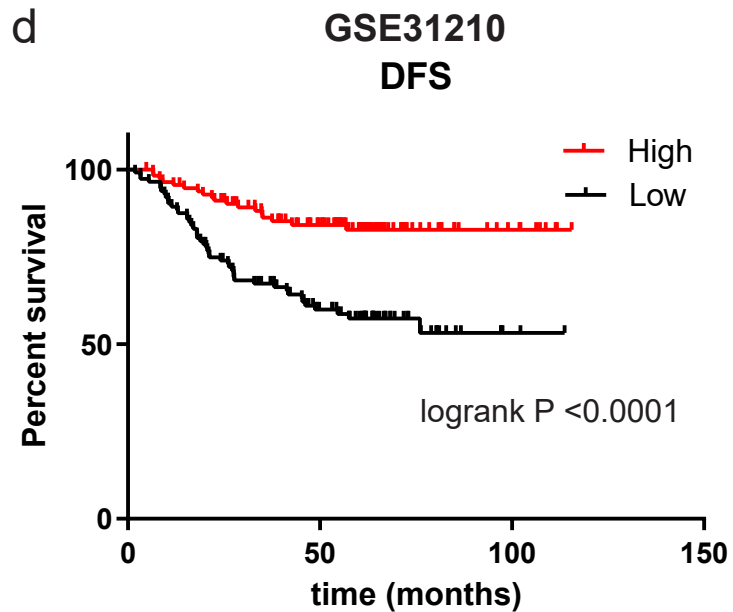

Supplement: Supplementary file 6 — Additional file 6: Figure S2. Impact of CLEC3B expression on OS and DFS in lung cancer patients from the GEO datasets. (a, b) OS survival curves and DFS survival curves of lung cancer patients in GSE30219. (c, d) and in GSE31210. OS, overall survival; DFS, disease-free survival. [file 12935_2020_1183_MOESM6_ESM.pdf]
