# Supplementary material for: CLEC3B as a potential diagnostic and prognostic biomarker in lung cancer and association with the immune microenvironment
Source: Cancer Cell Int. 2020 Apr 1;20:106. doi: 10.1186/s12935-020-01183-1 (PMC7110733; doi:10.1186/s12935-020-01183-1)
Supplement: Supplementary file 7 — Additional file 7: Table S4. Enrichment of GO in the CLEC3B high expression group of ADC [file 12935_2020_1183_MOESM7_ESM.docx]

**Table S4** Enrichment of GO in the CLEC3B high expression group of ADC

| **No.** | **Name** | **Size** | **ES** | **NES** | **NOM p-val** | **FDR q-val** |
| --- | --- | --- | --- | --- | --- | --- |
| 1 | GO_FLUID_TRANSPORT | 26 | 0.720 | 2.258 | 0.000 | 0.030 |
| 2 | GO_RENAL_ABSORPTION | 16 | 0.765 | 2.191 | 0.000 | 0.047 |
| 3 | GO_WATER_TRANSPORT | 19 | 0.744 | 2.115 | 0.000 | 0.104 |
| 4 | GO_COMPLEMENT_ACTIVATION | 67 | 0.630 | 2.049 | 0.000 | 0.123 |
| 5 | GO_C_C_CHEMOKINE_BINDING | 24 | 0.792 | 2.056 | 0.000 | 0.134 |
| 6 | GO_MULTIVESICULAR_BODY | 46 | 0.601 | 2.018 | 0.000 | 0.141 |
| 7 | GO_REGULATION_OF_SYSTEMIC_ARTERIAL_BLOOD_PRESSURE_BY_CIRCULATORY_RENIN_ANGIOTENSIN | 18 | 0.734 | 2.022 | 0.000 | 0.154 |
| 8 | GO_LAMELLAR_BODY | 17 | 0.804 | 2.056 | 0.000 | 0.167 |
| 9 | GO_CALCIUM_DEPENDENT_PROTEIN_BINDING | 62 | 0.499 | 1.966 | 0.000 | 0.189 |
| 10 | GO_NEGATIVE_REGULATION_OF_BLOOD_VESSEL_ENDOTHELIAL_CELL_MIGRATION | 32 | 0.618 | 1.954 | 0.000 | 0.200 |
| 11 | GO_CHEMOKINE_BINDING | 32 | 0.681 | 1.937 | 0.000 | 0.213 |
| 12 | GO_RENAL_SYSTEM_PROCESS | 113 | 0.459 | 1.908 | 0.000 | 0.227 |
| 13 | GO_DIPEPTIDASE_ACTIVITY | 15 | 0.635 | 1.913 | 0.000 | 0.229 |
| 14 | GO_BLOOD_COAGULATION_INTRINSIC_PATHWAY | 18 | 0.759 | 1.898 | 0.000 | 0.239 |
| 15 | GO_OXIDOREDUCTASE_ACTIVITY_ACTING_ON_PEROXIDE_AS_ACCEPTOR | 54 | 0.544 | 1.846 | 0.000 | 0.324 |
| 16 | GO_MEMBRANE_REPOLARIZATION | 44 | 0.526 | 1.849 | 0.000 | 0.329 |
| 17 | GO_PHOSPHOLIPID_CATABOLIC_PROCESS | 38 | 0.491 | 1.766 | 0.000 | 0.368 |
| 18 | GO_BILE_ACID_METABOLIC_PROCESS | 43 | 0.456 | 1.705 | 0.000 | 0.371 |
| 19 | GO_POSITIVE_REGULATION_OF_ION_TRANSPORT | 263 | 0.353 | 1.651 | 0.000 | 0.381 |
| 20 | GO_EXOPEPTIDASE_ACTIVITY | 110 | 0.415 | 1.793 | 0.000 | 0.388 |

Statistical data were performed by GSEA software.

**Abbreviations:** ES, enrichment score; FDR q‐val, false discovery rate q value; NES, normal enrichment score; NOM p‐val, nominal P‐value.
