# Supplementary material for: CLEC3B as a potential diagnostic and prognostic biomarker in lung cancer and association with the immune microenvironment
Source: Cancer Cell Int. 2020 Apr 1;20:106. doi: 10.1186/s12935-020-01183-1 (PMC7110733; doi:10.1186/s12935-020-01183-1)
Supplement: Supplementary file 8 — Additional file 8: Table S5. Enrichment of GO in the CLEC3B low expression group of ADC. [file 12935_2020_1183_MOESM8_ESM.docx]

**Table S5** Enrichment of GO in the CLEC3B low expression group of ADC

| **No.** | **Name** | **Size** | **ES** | **NES** | **NOM p-val** | **FDR q-val** |
| --- | --- | --- | --- | --- | --- | --- |
| 1 | GO_SPINDLE_MICROTUBULE | 54 | -0.755 | -2.338 | 0.000 | 0.001 |
| 2 | GO_MITOTIC_SPINDLE_ASSEMBLY | 53 | -0.718 | -2.262 | 0.000 | 0.001 |
| 3 | GO_MITOTIC_NUCLEAR_DIVISION | 279 | -0.601 | -2.271 | 0.000 | 0.001 |
| 4 | GO_MICROTUBULE_CYTOSKELETON_ORGANIZATION_INVOLVED_IN_MITOSIS | 123 | -0.679 | -2.317 | 0.000 | 0.001 |
| 5 | GO_CHROMOSOME_CENTROMERIC_REGION | 193 | -0.683 | -2.277 | 0.000 | 0.001 |
| 6 | GO_SISTER_CHROMATID_SEGREGATION | 180 | -0.676 | -2.285 | 0.000 | 0.001 |
| 7 | GO_DNA_DEPENDENT_DNA_REPLICATION | 146 | -0.716 | -2.281 | 0.000 | 0.001 |
| 8 | GO_DNA_REPLICATION | 271 | -0.647 | -2.257 | 0.000 | 0.001 |
| 9 | GO_MITOTIC_SPINDLE_ORGANIZATION | 102 | -0.698 | -2.350 | 0.000 | 0.002 |
| 10 | GO_CHROMOSOME_SEGREGATION | 304 | -0.641 | -2.282 | 0.000 | 0.002 |
| 11 | GO_SPINDLE_ORGANIZATION | 166 | -0.627 | -2.290 | 0.000 | 0.002 |
| 12 | GO_NUCLEAR_CHROMOSOME_SEGREGATION | 252 | -0.636 | -2.235 | 0.000 | 0.002 |
| 13 | GO_KINETOCHORE | 133 | -0.701 | -2.241 | 0.000 | 0.002 |
| 14 | GO_REGULATION_OF_DNA_REPLICATION | 106 | -0.651 | -2.245 | 0.000 | 0.002 |
| 15 | GO_MITOTIC_SISTER_CHROMATID_SEGREGATION | 148 | -0.675 | -2.233 | 0.000 | 0.002 |
| 16 | GO_SPINDLE_MIDZONE | 34 | -0.703 | -2.225 | 0.000 | 0.002 |
| 17 | GO_CELLULAR_RESPONSE_TO_HEAT | 132 | -0.561 | -2.208 | 0.000 | 0.002 |
| 18 | GO_REGULATION_OF_DNA_DEPENDENT_DNA_REPLICATION | 51 | -0.754 | -2.219 | 0.000 | 0.002 |
| 19 | GO_SPINDLE_ASSEMBLY | 105 | -0.618 | -2.209 | 0.000 | 0.002 |
| 20 | GO_CONDENSED_CHROMOSOME | 221 | -0.647 | -2.216 | 0.000 | 0.002 |

Statistical data were performed by GSEA software.

**Abbreviations:** ES, enrichment score; FDR q‐val, false discovery rate q value; NES, normal enrichment score; NOM p‐val, nominal P‐value.
