# Supplementary material for: CLEC3B as a potential diagnostic and prognostic biomarker in lung cancer and association with the immune microenvironment
Source: Cancer Cell Int. 2020 Apr 1;20:106. doi: 10.1186/s12935-020-01183-1 (PMC7110733; doi:10.1186/s12935-020-01183-1)
Supplement: Supplementary file 9 — Additional file 9: Table S6. Enrichment of GO in the CLEC3B high expression group of SCC. [file 12935_2020_1183_MOESM9_ESM.docx]

**Table S6** Enrichment of GO in the CLEC3B high expression group of SCC

| **No.** | **Name** | **Size** | **ES** | **NES** | **NOM p-val** | **FDR q-val** |
| --- | --- | --- | --- | --- | --- | --- |
| 1 | GO_CALCIUM_MEDIATED_SIGNALING | 214 | 0.585 | 2.509 | 0.000 | 0.000 |
| 2 | GO_SIDE_OF_MEMBRANE | 497 | 0.609 | 2.505 | 0.000 | 0.000 |
| 3 | GO_CLATHRIN_COATED_ENDOCYTIC_VESICLE | 60 | 0.730 | 2.465 | 0.000 | 0.000 |
| 4 | GO_EXTERNAL_SIDE_OF_PLASMA_MEMBRANE | 312 | 0.662 | 2.434 | 0.000 | 0.000 |
| 5 | GO_ADAPTIVE_IMMUNE_RESPONSE | 393 | 0.663 | 2.405 | 0.000 | 0.000 |
| 6 | GO_TERTIARY_GRANULE | 163 | 0.638 | 2.387 | 0.000 | 0.001 |
| 7 | GO_PHAGOCYTOSIS | 252 | 0.601 | 2.378 | 0.000 | 0.001 |
| 8 | GO_ENDOCYTIC_VESICLE | 293 | 0.563 | 2.363 | 0.000 | 0.001 |
| 9 | GO_LYMPHOCYTE_MEDIATED_IMMUNITY | 247 | 0.649 | 2.356 | 0.000 | 0.001 |
| 10 | GO_NEGATIVE_REGULATION_OF_IMMUNE_SYSTEM_PROCESS | 438 | 0.555 | 2.354 | 0.000 | 0.001 |
| 11 | GO_SECRETORY_GRANULE_MEMBRANE | 294 | 0.586 | 2.343 | 0.000 | 0.001 |
| 12 | GO_MACROPHAGE_ACTIVATION | 83 | 0.710 | 2.343 | 0.000 | 0.001 |
| 13 | GO_IMMUNE_RESPONSE_REGULATING_CELL_SURFACE_RECEPTOR_SIGNALING_PATHWAY | 385 | 0.564 | 2.340 | 0.002 | 0.001 |
| 14 | GO_REGULATION_OF_CALCIUM_MEDIATED_SIGNALING | 97 | 0.558 | 2.335 | 0.000 | 0.001 |
| 15 | GO_B_CELL_MEDIATED_IMMUNITY | 116 | 0.654 | 2.333 | 0.000 | 0.001 |
| 16 | GO_LEUKOCYTE_PROLIFERATION | 287 | 0.607 | 2.330 | 0.002 | 0.001 |
| 17 | GO_RESPIRATORY_BURST | 32 | 0.741 | 2.330 | 0.000 | 0.001 |
| 18 | GO_REGULATION_OF_IMMUNE_EFFECTOR_PROCESS | 390 | 0.581 | 2.328 | 0.000 | 0.001 |
| 19 | GO_REGULATION_OF_LIPOPOLYSACCHARIDE_MEDIATED_SIGNALING_PATHWAY | 25 | 0.741 | 2.326 | 0.000 | 0.001 |
| 20 | GO_REGULATION_OF_LEUKOCYTE_PROLIFERATION | 216 | 0.619 | 2.323 | 0.002 | 0.001 |

Statistical data were performed by GSEA software.

**Abbreviations:** ES, enrichment score; FDR q‐val, false discovery rate q value; NES, normal enrichment score; NOM p‐val, nominal P‐value.
