# Supplementary material for: CLEC3B as a potential diagnostic and prognostic biomarker in lung cancer and association with the immune microenvironment
Source: Cancer Cell Int. 2020 Apr 1;20:106. doi: 10.1186/s12935-020-01183-1 (PMC7110733; doi:10.1186/s12935-020-01183-1)
Supplement: Supplementary file 10 — Additional file 10: Table S7. Enrichment of GO in the CLEC3B low expression group of SCC. [file 12935_2020_1183_MOESM10_ESM.docx]

**Table S7** Enrichment of GO in the CLEC3B low expression group of SCC

| **No.** | **Name** | **Size** | **ES** | **NES** | **NOM p-val** | **FDR q-val** |
| --- | --- | --- | --- | --- | --- | --- |
| 1 | GO_CONDENSED_CHROMOSOME_CENTROMERIC_REGION | 117 | -0.613 | -1.983 | 0.004 | 0.034 |
| 2 | GO_TRANSCRIPTION_BY_RNA_POLYMERASE_I | 62 | -0.576 | -1.980 | 0.002 | 0.035 |
| 3 | GO_MITOTIC_SPINDLE_ASSEMBLY | 53 | -0.627 | -1.984 | 0.002 | 0.035 |
| 4 | GO_DNA_POLYMERASE_BINDING | 17 | -0.810 | -1.967 | 0.004 | 0.035 |
| 5 | GO_TRNA_TRANSPORT | 36 | -0.702 | -1.986 | 0.000 | 0.035 |
| 6 | GO_TERMINATION_OF_RNA_POLYMERASE_II_TRANSCRIPTION | 34 | -0.656 | -1.954 | 0.002 | 0.036 |
| 7 | GO_CHROMOSOME_CONDENSATION | 42 | -0.620 | -1.988 | 0.004 | 0.036 |
| 8 | GO_PRONUCLEUS | 16 | -0.758 | -1.972 | 0.000 | 0.036 |
| 9 | GO_RIBONUCLEOPROTEIN_COMPLEX_BIOGENESIS | 439 | -0.530 | -1.948 | 0.008 | 0.036 |
| 10 | GO_MITOCHONDRIAL_RNA_METABOLIC_PROCESS | 23 | -0.715 | -1.967 | 0.000 | 0.036 |
| 11 | GO_REGULATION_OF_SPINDLE_ASSEMBLY | 27 | -0.651 | -1.951 | 0.000 | 0.036 |
| 12 | GO_NCRNA_PROCESSING | 374 | -0.537 | -1.970 | 0.006 | 0.036 |
| 13 | GO_CHROMOSOME_LOCALIZATION | 75 | -0.555 | -1.949 | 0.000 | 0.036 |
| 14 | GO_U2_TYPE_CATALYTIC_STEP_2_SPLICEOSOME | 30 | -0.694 | -1.955 | 0.000 | 0.036 |
| 15 | GO_RAN_GTPASE_BINDING | 39 | -0.597 | -1.942 | 0.002 | 0.036 |
| 16 | GO_MATURATION_OF_LSU_RRNA | 20 | -0.745 | -1.961 | 0.000 | 0.036 |
| 17 | GO_DNA_CONFORMATION_CHANGE | 296 | -0.528 | -1.973 | 0.000 | 0.036 |
| 18 | GO_RNA_3_END_PROCESSING | 147 | -0.567 | -2.009 | 0.000 | 0.036 |
| 19 | GO_NUCLEAR_PORE | 85 | -0.565 | -2.012 | 0.000 | 0.036 |
| 20 | GO_MITOTIC_SISTER_CHROMATID_SEGREGATION | 148 | -0.571 | -1.944 | 0.002 | 0.037 |

Statistical data were performed by GSEA software.

**Abbreviations:** ES, enrichment score; FDR q‐val, false discovery rate q value; NES, normal enrichment score; NOM p‐val, nominal P‐value.
